# The Prevalence of Anti-Hexokinase-1 and Anti-Kelch-Like 12 Peptide Antibodies in Patients With Primary Biliary Cholangitis Is Similar in Europe and North America: A Large International, Multi-Center Study

**DOI:** 10.3389/fimmu.2019.00662

**Published:** 2019-04-03

**Authors:** Gary L. Norman, Anna Reig, Odette Viñas, Michael Mahler, Ewa Wunsch, Piotr Milkiewicz, Mark G. Swain, Andrew Mason, Laura M. Stinton, Maria Belen Aparicio, Maria Jose Aldegunde, Marvin J. Fritzler, Albert Parés

**Affiliations:** ^1^Department of Research and Development, Inova Diagnostics, San Diego, CA, United States; ^2^Liver Unit, Hospital Clínic, Institut D'Investigacions Biomèdiques August Pi i Sunyer, CIBERehd, University of Barcelona, Barcelona, Spain; ^3^Immunology Department, Hospital Clínic, Centre Diagnòstic Biomèdic, Institut D'Investigacions Biomèdiques August Pi i Sunyer, Barcelona, Spain; ^4^Translational Medicine Group, Pomeranian Medicine University, Szczecin, Poland; ^5^Liver and Internal Medicine Unit, Department General, Transplant and Liver Surgery, Medical University of Warsaw, Warsaw, Poland; ^6^Cumming School of Medicine, University of Calgary, Calgary, AB, Canada; ^7^Division of Gastroenterology (Liver Unit), University of Alberta, Edmonton, AB, Canada; ^8^Laboratorio Autoimmunidad, Hospital Universitario de Salamanca, Salamanca, Spain

**Keywords:** primary biliary cholangitis, autoantibodies, anti-mitochondrial antibodies, AMA-negative, hexokinase-1, kelch-like 12

## Abstract

Primary biliary cholangitis (PBC), formerly known as primary biliary cirrhosis, is present worldwide. Autoantibodies, in particular anti-mitochondrial antibodies (AMA) detected by indirect immunofluorescence assays or newer solid phase immunoassays can detect most, but not all individuals with PBC. Detection of antibodies to the anti-nuclear antigens sp100 and gp210 can identify additional PBC patients, but some seronegative patients remain, often resulting in delayed diagnosis and treatment. Antibodies to kelch-like 12 (KLHL12) and hexokinase 1 (HK-1) were recently identified as new biomarkers for PBC and notably identify patients who are negative for conventional autoantibodies. To become globally adopted, it is important to validate these new biomarkers in different geographic areas. In the present study we evaluated the prevalence of anti-KLHL12 (measured by a KLHL12-derived peptide referred to as KL-p) and anti-HK-1 antibodies by ELISA at five sites within Europe and North America and demonstrated the presence of these antibodies in patients with PBC in all geographies.

## Introduction

For over 50 years, the detection of anti-mitochondrial antibodies (AMA) on rodent tissue sections has remained the primary diagnostic biomarker for primary biliary cholangitis (PBC) in many laboratories, and when performed and interpreted at expert centers, the assay is highly sensitive and specific for PBC (1–3). It is clear however, that up to 15% of patients with clinically-proven PBC are AMA-negative by indirect immunofluorescence (IIF) (4–7). Using recombinant proteins such as M2 (MIT3), which incorporates the three immunodominant epitopes recognized by AMA, namely the E2 subunits of pyruvate dehydrogenase complex (PDC-E2), the branched chain 2-oxo-acid dehydrogenase complex (BCOADC-E2), and the 2-oxo-glutareate dehydrogenase complex (OGDC-E2), can result in increased sensitivity and the ability to detect additional, but still not all, AMA-negative PBC patients (4, 8, 9). While additional AMA-negative PBC patients can be identified by the presence of anti-sp100 corresponding to the multiple nuclear dot pattern by IIF on HEp-2 cells (AC-6 according to International Consensus of ANA patterns, ICAP), anti-gp210 punctate nuclear envelope by IIF on HEp-2 cells (AC-12 according to ICAP), and anti-p62 antibodies, some PBC patients remain seronegative, potentially resulting in delayed diagnosis and treatment (10–14).

Autoantibodies to kelch-like 12 protein (KLHL12) and to hexokinase 1 (HK-1) were recently identified as novel biomarkers in patients with PBC (15, 16). In a cohort of 366 patients with PBC, ~40 and 45% of 277 AMA-positive patients were positive for anti-KLHL12 or anti-HK-1 antibodies respectively, while in 89 AMA-negative patients 53 and 42% were positive for anti-KLHL12 or anti-HK-1 antibodies, respectively. The specificities of both antibodies was 96–97% (15).

KLHL12 is part of a large, evolutionarily conserved superfamily consisting of 66 KLHL genes (17). The various Kelch proteins appear to be involved in multiple cellular functions including cell structure, cellular communication, transcriptional regulation, collagen export, and ubiquitination of proteins through interaction with the E3-ligase cullin (17, 18). HK-1 is an enzyme which localizes to the outer membrane of mitochondria and phosphorylates glucose to yield glucose-6-phosphate, as well as modulating cellular susceptibility to apoptosis (19).

Although difficult to clearly establish, it appears that the prevalence of PBC is increasing worldwide (20–22). While AMA has a similar prevalence in PBC patients from different geographies, the prevalence of anti-KLHL12 and anti-HK-1 antibodies in different geographic areas has not been reported. To address this issue, we examined PBC sera collected from patients in Eastern Europe, Western Europe, and Canada for the presence of anti-KLHL12 and anti-HK-1 antibodies.

## Materials and Methods

Sera from a total of 487 patients with clinically documented PBC or PBC/AIH overlap diagnosed according to European Association for the Study of the Liver (EASL) guidelines were collected at five expert clinical sites (Barcelona, Spain; Salamanca, Spain; Calgary, Canada; Edmonton, Canada; Warsaw, Poland) and tested for the presence of autoantibodies to HK-1 and a KLHL12-derived immunodominant peptide, referred to as “KL-p,” using research use only ELISA kits (QUANTA Lite®, Inova Diagnostics, San Diego, CA). All sera were also tested for anti-M2 (MIT3) antibodies (subsequently referred to as anti-MIT3 for simplicity) by ELISA (QUANTA Lite® ELISA, Inova Diagnostics, San Diego, CA). Specificity was assessed by testing 127 sera from patients without PBC, including patients with primary sclerosis cholangitis (PSC, *n* = 41), autoimmune hepatitis (AIH, *n* = 20), AIH/PSC overlap (*n* = 12), various infectious diseases (*n* = 20), colorectal cancer (*n* = 14), and healthy controls (*n* = 20).

Differences between the five cohorts was assessed by one-way analysis of variance (ANOVA) using Krustal-Wallis test and between specific geographic chorts using Mann-Whitney non-parametric two-tailed *t*-test (Graphpad ver 5.03, San Diego, CA). *P*-value <0.05 was considered significant.

## Results

Testing for anti-MIT3 antibodies showed that each cohort had different frequencies of anti-MIT3 negative individuals (Calgary, 7.2%; Barcelona, 12.9%, Edmonton, 16.3%; Salamanca, 23.8%, and Warsaw, 36.6%) (*p* = 0.0002). Overall anti-HK-1 antibodies were found in 36.6–52.4% and anti-KL-p antibodies in from 22.0–33.3% of the cohorts. The specific prevalence of anti-HK-1 and KL-p antibodies at each site is summarized in Table 1.

**Table 1:** Frequency of anti-HK-1, anti-KL-p, and anti-HK-1 and/or anti-KL-p in each geographic cohort and in a combined cohort including all patients.

		**Anti-HK-1+**			**Anti-KL-p+**			**Anti-HK-1+ and/or KL-p+**		
	**Total Cohort *N* =**	**No. (%) MIT3+**	**No. (%) MIT3–**	***P*- value**	**No. (%) MIT3+**	**No. (%) MIT3–**	***P*-value**	**No. (%) MIT3+**	**No. (%) MIT3–**	***P*-value**
Barcelona	224	75/195 (38.5)	10/29 (34.5)	0.8379	49/195 (25.1)	3/29 (10.3)	0.0991	103/195 (52.8)	13/29 (44.8)	0.4341
Calgary	97	48/90 (53.3)	1/7 (14.8)	0.0592	23/90 (25.6)	2/7 (28.6)	1.000	59/90 (65.6)	3/7 (42.9)	0.2486
Edmonton	104	45/87 (51.7)	2/17 (11.8)	**0.0028**	22/87 (25.2)	2/17 (11.8)	0.3476	54/87 (62.1)	3/17 (17.6)	**0.0014**
Warsaw	41	12/26 (46.2)	3/15 (20.0)	0.1772	6/26 (23.1)	3/15 (20.0)	1.000	15/26 (57.7)	5/15 (33.3)	0.1971
Salamanca	21	9/16 (56.3)	2/5 (40.0)	0.6351	3/16 (18.8)	4/5 (80.0)	**0.0251**	9/16 (56.2)	4/5 (80.0)	0.6065
COMBINED	487	189/414 (45.7)	18/73 (24.7)	**0.0008**	103/414 (24.9)	14/73 (19.2)	0.3725	240/414 (58.0)	28/73 (38.4)	**0.0022**

*P-values <0.5 are considered significant and indicated by bold type*.

As a result of the apparent heterogeneity of the cohorts, we proceeded to examine the performance of the two biomarkers after specimens were stratified by anti-MIT3 positivity. Anti-HK-1 antibody positivity in anti-MIT3-positive individuals ranged from 38.5–56.3% as detailed in Table 1. While the groups were statistically different overall (ANOVA *p* = 0.0006), there was no difference between some of the sites, for example Barcelona and Edmonton (*p* = 0.8169). When all specimens from the five cohorts were combined, anti-HK-1 antibody was positive in 45.7% (189/414) of the anti-MIT3-positive specimens, with a specificity of 94.5% (120/127). Of the seven non-PBC specimens that tested positive, four had a diagnosis of AIH, two colorectal cancer, and one was an apparently healthy control. The likelihood ratio (LR)+, LR-, positive predictive value (PPV), negative predictive value (NPV), and Odds Ratio (OR) of anti-HK-1 antibodies for the combined cohort of all anti-MIT3-positive specimens was 8.28, 0.58, 0.96, 0.35, and 14.40.

Anti-KL-p antibody positivity in the anti-MIT3-positive specimens ranged from 18.8–25.6% (Table 1). Overall, when all specimens were combined, anti-KL-p antibody was positive in 24.9% (103/414) of the anti-MIT3-positive specimens with a specificity of 95.3% (121/127). As with anti-HK-1 antibodies, while the groups were statistically different overall (ANOVA *p* < 0.0001), several showed no statistical difference, again for example Barcelona-Edmonton, Barcelona-Warsaw, Edmonton-Warsaw (*p* = 0.9973, 0.4333, and 0.0868 respectively). The LR+, LR-, PPV, NPV, and OR for anti-KL-p antibodies for the combined cohort of anti-MIT3-positive specimens was 4.60, 0.79, 0.96, 0.18, and 5.80.

Examination of anti-MIT3-negative PBC patients in the five cohorts showed a prevalence of anti-HK-1 antibodies ranging from 11.8–40.0% and anti-KL-p antibodies from 10.3 to 80.0% (Table 1). Among all anti-MIT3-negative specimens from the 5 sites, anti-HK-1 antibodies were found in 18/73 (24.7%) and anti-KL-p in 14/73 (19.2%) of the patients. Employing both markers to examine the anti-MIT3-negative patients increased the sensitivity to 38.4% (28/73) with a small decrease in specificity to 91.3%.

**Figure 1:**
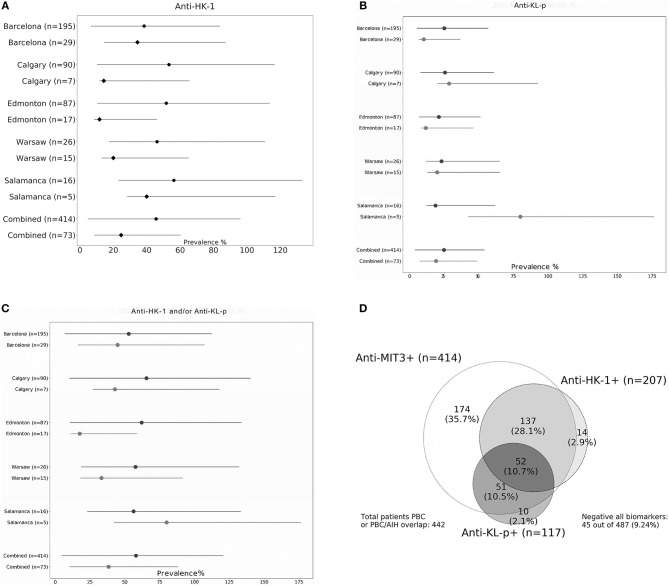
Prevalence of anti-HK-1 **(A)**, anti-KL-p **(B)**, and combined anti-HK-1/KL-p antibodies **(C)** in cohorts from five individual sites and combined cohort consisting of all specimens tested (both anti-MIT3-positive and negative PBC specimens). Frequency in anti-MIT3-negative patients indicated by black diamonds and frequency in anti-MIT3-positive patients by black dots. Venn diagram **(D)** showing overlap of anti-MIT3, anti-HK-1, and anti-KL-p antibodies. The cohort (*n* = 487) contains patients with primary biliary cholangitis (PBC, *n* = 474), PBC-autoimmune hepatitis (AIH) overlap (*n* = 13), including both anti-MIT3-positive (*n* = 414) and anti-MIT3-negative (*n* = 73) PBC samples.

The overlap of anti-MIT3, anti-HK-1, and anti-KL-p antibodies is illustrated using a Venn diagram in Figure 1. The addition of anti-HK-1 and anti-KL-p antibody testing to anti-MIT3 antibody results increased the sensitivity from 85.0% for anti-MIT3 alone to 90.8% for the combination of anti-MIT3, anti-HK-1, and anti-KL-p for the total study cohort.

## Discussion

The incidence of PBC, like many other autoimmune diseases, appears to be increasing worldwide (20–22). Environmental factors have been suggested as one potential contributing cause of the increasing incidence of PBC (3). In addition, better diagnostics and better recognition of the disease, especially during the often long asymptomatic phase, are also likely involved in the apparent increased prevalence (23, 24). The availability of new therapeutics such as obeticholic acid provides an additional rationale to identify patients with PBC early in the disease course, so that treatment and monitoring that might slow, or ideally prevent progression can be instituted (22). New biomarkers to help identify AMA or anti-MIT3-negative PBC patients who may otherwise remain unrecognized, are particularly important to help achieve this goal.

Despite the regular description of new biomarkers in the literature, only a few contribute additional actionable clinical value and are incorporated into routine clinical practice. We have previously shown that anti-HK-1 and anti-KL-p antibodies are present in both AMA-positive and, importantly, AMA-negative patients (15). Our current results demonstrate that the general prevalence of these autoantibodies is similar in 5 different sites examined from North America and Europe. The pooled prevalence of anti-HK-1 antibodies was 45.7% in anti-MIT3-positive patients and 24.7% in anti-MIT3-negative patients. Anti-KL-p antibodies were detected in 24.9% of the anti-MIT3-positive patients and 19.2% of the anti-MIT3-negative patients. Combined testing for both antibodies found 58.0% of the anti-MIT3-positive and 38.4% of the anti-MIT3-negative patients seropositive for one or both of the new antibodies. The new biomarkers therefore may help to close the serological gap in PBC patients. The prevalence of the markers appeared more similar in the anti-MIT3-positive patients from the different sites compared to the anti-MIT3-negative patients. This is reasonable since the anti-MIT3-positive patients are likely a more clinically homogeneous group compared to the anti-MIT3-negative group, which may have a more heterogeneous phenotype. The small sample size of several of the cohorts is also a clear limitation of the study and likely contributes to some of the perceived differences between cohorts. The higher prevalence of anti-HK-1 antibodies compared to anti-KL-p antibodies could be related to the presence of HK-1 on the mitochondrial membrane, similar to other PBC-related antigens. In contrast, explanation why antibodies to KLHL12 are increased in patients with PBC remains intriguing, but still unclear at present.

Early treatment of patients with PBC appears beneficial and, therefore, addition of anti-KL-p and anti-HK-1 autoantibody testing has the potential to decrease delays in diagnosis and treatment (22). Although we did not examine the prevalence of the antibodies in Asian and South American populations, these studies are currently being organized. A key question remaining is whether, in addition to their value in diagnosis, these antibodies can be shown to be associated with particular clinical phenotypes or outcomes. Preliminary results, so far only presented in abstract form, suggest this may be the case for anti-HK-1 antibodies, where the presence of these antibodies at diagnosis was associated with significantly increased levels of alkaline phosphatase, alanine aminotransaminase, and gamma-glutamyl transpeptidase compared to anti-HK-1 negative patients, as well as with shorter transplant-free survival time (25). These results are potentially of important clinical interest, however additional validation and studies are now needed to confirm their significance.

## Author Contributions

GN, MM, AP, MS, AM, PM, MF, and AR contributed to the design of the study. OV, EW, MBA, MJA, and LS contributed to the acquisition and analysis of data. GN wrote the manuscript. All authors reviewed and approved the publication of the content.

### Conflict of Interest Statement

GN, MM are employees of Inova Diagnostics, Inc. MF and AP have received speaking and consulting honoraria from Inova Diagnostics, Inc. and Werfen International. The remaining authors declare that the research was conducted in the absence of any commercial or financial relationships that could be construed as a potential conflict of interest.
